# Assessment of gubernacular canal frequency with CBCT in a group of Turkish population

**DOI:** 10.1186/s12903-023-03608-5

**Published:** 2023-11-13

**Authors:** Mehmet Emin Dogan, Nurbanu Uluısık, Mehmet Sinan Dogan

**Affiliations:** 1 Faculty of Dentistry, Department of Dentomaxillofacial Radiology, Harran University, Sanliurfa, Turkey; 2 Faculty of Dentistry, Department of Pediatric Dentistry, Harran University, Sanliurfa, Turkey

**Keywords:** Tooth eruption, Cone beam computed tomography, Impacted, Gubernacular canal

## Abstract

**Objective:**

The aim of this study is to investigate the frequency of the gubernacular canal observed on cone-beam computed tomography (CBCT) images in a group of Turkish populations according to age and gender.

**Materials and methods:**

CBCT images of 500 cases were evaluated retrospectively, and a total of 117 impacted or erupting teeth were included in the study, and the presence of gubernacular canal was recorded according to age, gender and eruption status of the tooth. SPSS program was used for statistical analysis.

**Results:**

The mean age of 117 buried/continuing patients was 17 ± 15.32, 55 (47%) were female and were 62 (53%) male patients. Presence of gubernacular canal was observed in 91 (77.8%) cases and this duct was not detected in 26 (22.2%) cases. While 40.2% of female were found to have canals, 37.6% of male had canals. When the presence of canals was examined, the presence of canals was found to be significantly higher in the erupting teeth. A significant difference was found when analyzed according to the mean age.

**Conclusions:**

CBCT is a useful method of detecting the gubernacular canal. The presence of gubernacular canal was significantly higher in erupting teeth.

## Introduction

Tooth eruption is a complex, lifelong event that starts from the formation of the tooth germ as a result of a series of developmental events [1]. This process also means the transition of without function teeth in the alveolar bone to a useful state. The gubernaculum dentis, also known as the gubernacular canal (GC), is filled with fibrous connective tissue containing nerves, vessels, and lymphatic canals [2]. GC tissue also contains many chemical signaling mediators such as EGF4 which has the ability to induce osteoclast production. This causes resorption of the bone and thus the canal widens as the tooth approaches the alveolar bone [3].

The gubernacular cord and canal were first described by John Hunter [4] when he observed a contact among the growing tooth and gingiva, and Louis-Charles Malassez [5] supported this description by microscopic studies observing epithelial cell remnants of the dental lamina, together with fibers in the canal connecting the erupting tooth with the gingiva [3]. The GC is a intraosseous canal expressed as a minimal aperture on the lingual or palatal plane of erupting teeth. Some researchers have reported that this structure is only in primary teeth [6]. However, this view was quickly disproved when Scott [7] demonstrated the connection of the follicles of permanent molars without deciduous tooth precursors with the covering mucosa, the so-called “molar gubernacular cords”. This was a milestone as it implied that all erupting permanent teeth would have an eruption pathway up to the oral mucosa, which guides them into the eruption position [3].

It has been observed that the find rate of GCs on imaging differ at different rates in the literature, with a high rate in normally erupting teeth, followed by impacted teeth, and the lowest finding rate in delayed eruption teeth [8].

As the teeth erupt, resorption of the GC start. On radiographic images, the gubernacular canal is defined as a radiolucent canal with cortical borders attached to the tooth follicle. Since GC has a thin anatomical structure, it is hard to visualize on 2D radiographs [9]. GC can also be mistaken for an alimentary canal, an accessory arm of the mandibular canal, or a fistula tract on radiographs due to its location in the jaw [9]. The presence and imaging properties of this canal have been more extensively analyzed in recent studies using cone-beam computed tomography (CBCT) and multidetector computed tomography (MDCT) [10].

The use of CBCT in dentistry has increased in recent years owing to its advantages like low radiation dose (compared to MDCT), high bone resolution, ease of use and accessibility. It is also one of the safest and most useful methods of examining the jaws [2]. Volumetrical datum let accurate description of tooth morphology and its relation to surrounding structures, thus aiding clinical judgment making [11].

As far as we look at the literature, no study has been found about the frequency of GC in the Turkish population before. The objective of this study is to investigate the existence of GC in erupted and impacted teeth in the Turkish population with CBCT.

## Materials and methods

**Fig. 1:**
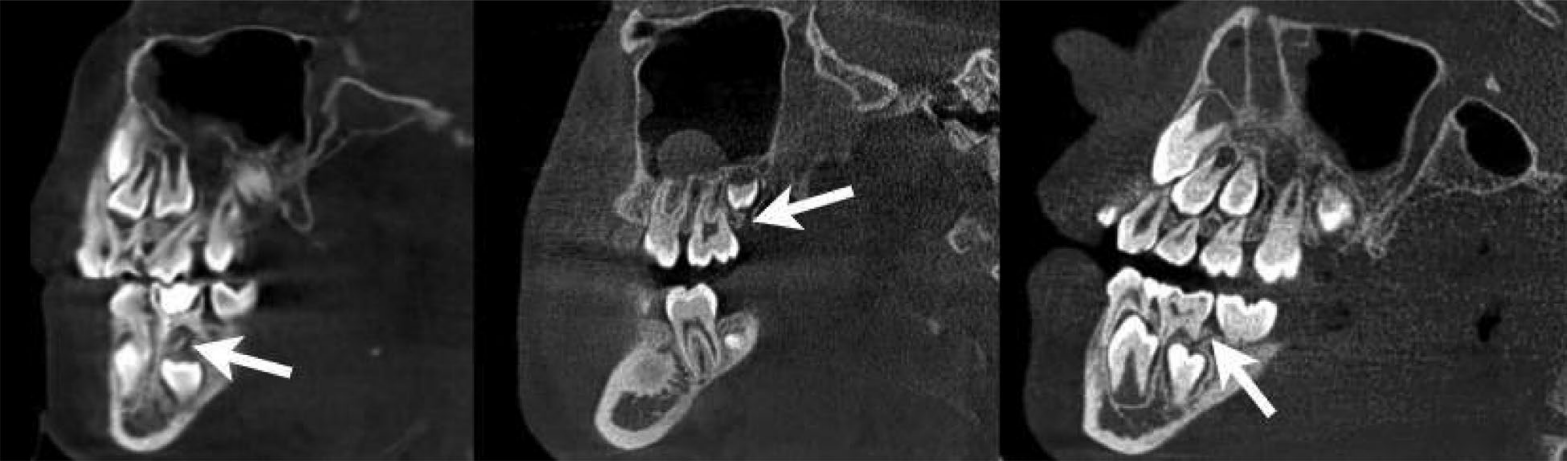
Sagittal image on CBCT of the gubernacular canal (marked by arrow) in posterior teeth

**Fig. 2:**
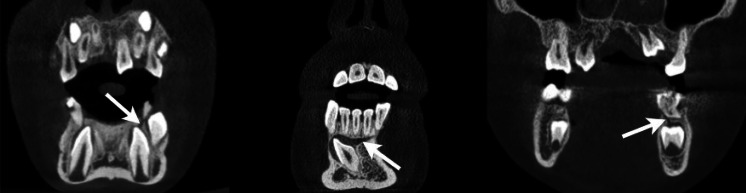
Coronal image on CBCT of the gubernacular canal (marked by arrow) in erupting teeth

In order to collect data for this study, 500 CBCT images in the archive of the Department of Dentomaxillofacial Radiology, Faculty of Dentistry, Harran University were scanned. Images with impacted or erupting teeth were included in the study. Patients with any pathology in the examination area, dental anomalies, edentulous patients and patients with image artifacts were excluded. Castellini X Radıus Trıo 3D (Imola, ITALY) tomography device with 90 kvp, 13–16 mA, 13 × 16, 13 × 10 cm fov parameters was used to obtain the images. The gubernacular canal was examined in multiplanar images reconstructed using IRYS software program (Figs. 1 and 2). All evaluations were performed by a dentomaxillofacial radiologist (M.E.D) with 5 years of experience. Patients’ age, gender, presence of gubernacular canal in erupting or impacted teeth were recorded.

### Statistical analysis

Data analysis was done with SPSS package program version 25 (Armonk, NY, IBM). Shapiro-Wilks test was used to check the normal dispersion of the data. Descriptive statistics were used to obtain values such as number (N), percentage (%) and mean. Chi-square test was used to examine the relation among categorical factors. Kruskall Wallis test was used for comparison between age groups and Mann Whitney U test was used for pairwise comparison. Kappa analysis was performed for intraobserver agreement. P < 0.05 was acknowledged as importance level.

## Results

In this research, 500 CBCT were screened and 55 (47%) female and 62 (53%) male with a mean age of 17 ± 15.32 years were inclusive. Intraobserver agreement was found to be excellent (0.90). Presence of gubernacular canal was observed in 91 (77.8%) of the cases. In 26 (22.2%) cases, this canal was not defined. As 68.4% of the cases were erupting teeth, 31.6% were impacted teeth. In females, the canal was detected in 47 (40.2%) images, while it was not detected in 8 (6.8%) images. In males, canals were present in 44 (37.6%) images, but were not detected in 18 (15.4%) images. When the existence of the canal was evaluated in accordance with gender, no statistically significant difference was observed (Table 1). Of the 80 (68.4%) erupting teeth, 70 (87.5%) had a gubernacular canal, while 10 (12.5%) did not. Of the 37 impacted teeth, 21(56.8%) had a canal, while 16 (43.2%) did not. When the presence of the canal was analyzed according to the eruption status of the tooth, the existence of the canal was statistically importantly higher in the erupting teeth (p < 0.05) (Table 2). When the distribution of the data considering the mean age was analyzed, a statistically meaningful difference was detected (p < 0.05). In the presence of GC, the average age was found to be 19.70 ± 12.99, and in the absence of GC, the average age was 34.50 ± 17.44. The distribution of GC presence according to age groups is shown in Table 3. The presence of GC was found to be significantly higher in individuals under 9 years of age than in individuals over 50 years of age. Significantly higher GC was observed in the 10–19 age group than in the 40–49 and over 50 age groups (p < 0.001).

**Table 1:** Distribution of the GC according to gender and eruption status

		Gender		Total	P value
		Female	Male		
		N (%)	N (%)		
Canal	Available	47 (40.2)	44 (37.6)	91 (77.8)	0.060
	None	8 (6.8)	18 (15.4)	26 (22.2)	
Total		55 (47)	62 (53)	117(100)	
Eruption Status	Erupting	37 (31.6)	43 (36.8)	80 (68.4)	0.809
	İmpacted	18 (15.4)	19 (16.2)	37 (31.6)	

**Table 2:** Distribution of the presence of GC according to the eruption status

		Eruption Status		Total	P value
		Erupting	İmpacted		
Canal	Available	70 (87.5%)	21 (56.8%)	91 (77.8%)	0.001*
	None	10 (12.5%)	16 (43.2%)	26 (22.2%)	
Total		80 (100%)	37 (100%)	117 (100%)	

* p < 0.05

**Table 3:** Distribution of GC presence according to age groups

Age Group	Canal		Total	P value
	Available	None		
	N (%)	N (%)		
≤ 9	8 (6.8)	1(0.09)	9 (7.7)	0.001*
10–19	55 (47.0)	7 (6.0)	62 (53.0)	
20–29	13(11.1)	3 (2.6)	16 (13.7)	
30–39	8 (6.8)	4 (3.4)	12 (10.2)	
40–49	4 (3.4)	5 (4.3)	9 (7.7)	
**≥** 50	3 (2.6)	6 (5.1)	9 (7.7)	
Total	91 (77.8)	26 (22.2)	117 (100)	

*p < 0.05

## Discussion

The GC is the anatomical eruption pathway linking the dental lamina of an unerupted tooth to the gingiva, found only in cancellous bone unrelated to the cortex. The GC can be observed radiographically in three-dimensional imaging [8]. In teeth on normal eruption, they appear as a radiolucent cortical limited pathway on CBCT continuing with the erupting tooth follicle on frontal and sagittal cross-sectional views. On axial images, they seem as low-density circular fields located upon the lingual/palatinal party of the deciduous tooth. The length of the GC can be recognized on coronal and sagittal planes. The width of the GC will vary with age because the erupting teeth will have a smaller canal, while the erupting tooth’s dental follicle will not be visible as it fuses with the alveolar crest [12]. Although GC is observed in radiography as a canal with 1–3 mm thickness, it is frequently hard for clinicians to diagnose on radiographs [10, 13]. However, with CBCT, the gubernacular canal can be visualized without any distortion or overlapping of anatomical structures and with minimal artifact formation [14]. In the clinical signifiance of the gubernacular canal, since the gubernacular canal of impacted teeth surrounded by bone tissue is less common, the presence of the canal in the treatment of the edentulous cavity protection with the application of placeholders shows us that the possibility of tooth eruption is high. With space maintainers, the arch length can be maintained and problems such as crowding, ectopic eruption and midline shift can be prevented [15, 16]. In addition, monitoring of this canal in clinical practice is important because it shows a high eruption rate that can guide the decision of whether to perform stop orthodontic treatment or surgical extraction of teeth with position and eruption anomalies [11, 17]. In its absence, a space maintainer should be used to preserve the size of the arch, and then orthodontic extrusion should be performed [18]. When the gubernacular canal is examined in teeth that do not erupt during the natural eruption period, it is known that eruption is delayed when the canal is absent or has an abnormal slope [19]. Therefore, this will affect the time the space maintainers stays in the mouth.

Koç et al. [10] examined the dispersion of GCs in impacted teeth in accordance with age groups and found a statistically significant difference. Although there are proportional differences, the decrease in the presence of GC as age increases is similar in our study. Absence of the GC may indicate a high risk of abnormal eruption and impaction of the involved tooth.

Araujo et al. [11] examined the gubernacular canal of 159 patients, including 423 teeth with normal eruption, 140 impacted teeth and 35 teeth with delayed eruption on CBCT images, found a canal observing rate of 90.6%. In addition, in line with these rates, the canal detection rate was calculated as the highest in normal erupted teeth, then impacted and the lowest in delayed erupted teeth, respectively. In our study, GC was observed in 72.6% of 117 patients. Araujo et al. [11] found that the detection rates of the GC were higher in normal erupted and impacted teeth in the primary stages of tooth constitution.

Furthermore, to differentiate the GC from alveolar bone resorption, teeth should be carefully evaluated in three reconstruction planes and the canal should be identified in at least two planes [11]. In our study, sagittal and transverse sections were predominantly examined, but it is useful to examine the canine teeth in coronal section.

Kaplan et al. [2] in their study in a sample with a mean age of 23.8 ± 11.6 years, they found the incidence of GC to be 31.7%. However, they reported that there was no significant difference according to age. In our study, a statistically significant difference was found according to age distribution.

Ugurlu et al. [20] evaluated the existence and typical of the GC in CBCT images of 231 patients with a mean age of 28.0 ± 0.90 years according to gender, age. While 31% (N:146) of the 471 teeth examined had canals, 69% (N:325) did not have canals. These values are different from our study; the lower incidence of the GC in this study can be explained by the higher mean age of the patients included, because the presence of GC decreases with age.

The limitations of this study are that since it was a retrospective study, information about the patients’ systemic diseases could not be obtained, pathological conditions that prevented tooth eruption could not be evaluated, and the presence of the canal became difficult to detect by including the abnormally positioned third molar tooth in the study. Data on the clinical prognosis of impacted/erupting teeth that could not be followed for eruption could not be obtained. Future studies are recommended to evaluate the gubernacular canal in a larger sample and in different jaws, including different tooth groups, classifying the canal direction.

## Conclusions

CBCT is a beneficial method for the find of the GC. In this study, the GC was observed more in erupting teeth. A significant difference was obtained according to the mean age and the probability of seeing the gubernacular canal decreases as the age increases. The presence or absence of GC is important in deciding on the treatment to be performed regarding the eruption of the tooth.

## Data Availability

The datasets generated or analyzed during the current study are available from the corresponding author upon reasonable request.
